# Case Finding and Medical Treatment of Type 2 Diabetes among Different Ethnic Minority Groups: The HELIUS Study

**DOI:** 10.1155/2017/9896849

**Published:** 2017-01-05

**Authors:** Marieke B. Snijder, Charles Agyemang, Ron J. Peters, Karien Stronks, Joanna K. Ujcic-Voortman, Irene G. M. van Valkengoed

**Affiliations:** ^1^Department of Public Health, Academic Medical Center, Amsterdam, Netherlands; ^2^Department of Cardiology, Academic Medical Center, Amsterdam, Netherlands; ^3^Public Health Service of Amsterdam, Amsterdam, Netherlands

## Abstract

*Aims*. Prevention of diabetes complications depends on the level of case finding and successful treatment of diabetes, which may differ between ethnicities. Therefore, we studied the prevalence by age, awareness, treatment, and control of type 2 diabetes, among a multiethnic population.* Methods*. We included 4,541 Dutch, 3,032 South-Asian Surinamese, 4,109 African Surinamese, 2,323 Ghanaian, 3,591 Turkish, and 3,887 Moroccan participants (aged 18–70 y) from the HELIUS study. The prevalence of diabetes was analysed by sex, ethnicity, and 10-year age groups. Ethnic differences in the prevalence, awareness, treatment, and control of diabetes were studied by logistic regression.* Results*. From the age of 31–40 years and older, the prevalence of diabetes was 3 to 12 times higher among ethnic minority groups than that among the Dutch host population. Awareness and medical treatment of diabetes were 2 to 5 times higher among ethnic minorities than that among Dutch. Among those medically treated, only 37–53% had HbA1c levels on target (≤7.0%); only Dutch men had HbA1c levels on target more often (67%).* Conclusions*. Our results suggest that the age limit for case finding among ethnic minority groups should be lower than that for the general population. Importantly, despite higher awareness and treatment among ethnic minorities, glycemic control was low, suggesting a need for increased efforts to improve the effectiveness of treatment in these groups.

## 1. Introduction

People with diabetes are at increased risk for cardiovascular disease and its related complications. Glycemic control may reduce this risk [1–4], but this largely depends on the level of case finding and adequate care. In the USA, the awareness, treatment, and control of diabetes have been shown to increase over the last decades, suggesting overall improvements in case finding and health care [5, 6]. However, ethnic differences in awareness and glycemic control remained, with lower awareness among Hispanic and Asian participants compared with African Americans and White Americans and less glycemic control among African Americans and Hispanic Americans, as compared with White Americans [5–7].

In Europe, a higher prevalence of diabetes is found among ethnic minority groups, such as African, Turkish, Moroccan, and particularly South-Asian origin groups, as compared with European host populations [8–13]. Data on awareness, treatment, and control among these groups, however, are very limited and the results are inconsistent. In contrast to the USA, the awareness seems to be higher among African and South-Asian ethnic minority groups as compared with the European host population [14–16]. For example, in 2003, 78% of South-Asians and Africans living in the Netherlands were aware of their diabetes, as compared with 58% among Dutch population [15]. Consistent with results from the USA, however, studies from the UK have also shown poorer diabetes control among African and South-Asian ethnic minority groups [17–19]. Information on awareness, treatment, and glycemic control among other large ethnic minority groups, such as Turkish and Moroccan groups, is currently lacking.

Diabetes appears to develop at a younger age in some ethnic minority groups, such as South-Asians, Africans, and Turkish and Moroccan origin groups, as compared to the host population [11, 15, 17, 19]. This may have consequences for the age at which case finding for diabetes should start among some ethnic groups. If the prevalence of diabetes is already substantially higher at a younger age among ethnic minority groups, case finding should also start at younger age. However, most previous studies had a limited age range or numbers were too small to adequately stratify by age.

Therefore, the aim of this study was to gain insight into both the age-specific prevalence of diabetes and the current levels of awareness, medical treatment, and glycemic control, among different ethnic groups. Results of this study may facilitate case finding and help the development of better guidelines for prevention and health care of diabetes.

## 2. Materials and Methods

### 2.1. Study Population

The HEalthy LIfe in an Urban Setting (HELIUS) study is a multiethnic cohort study conducted in Amsterdam which has been described in detail elsewhere [20]. In brief, baseline data collection took place in 2011–2015 and included people aged 18 to 70 years from six ethnic groups living in Amsterdam, that is, those of Dutch, South-Asian Surinamese, African Surinamese, Ghanaian, Moroccan, and Turkish origin. Participants were randomly, stratified by ethnicity, sampled from the municipal register. Data were collected by questionnaire and a physical examination in which biological samples were also obtained. The HELIUS study is conducted in accordance with the Declaration of Helsinki and has been approved by the AMC Ethical Review Board. All participants provided written informed consent.

**Figure 1:**
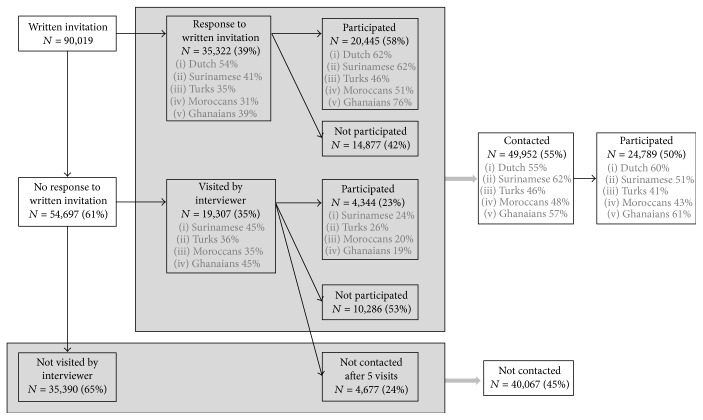
Flowchart of recruitment for the HELIUS study.

A flowchart of the recruitment for the HELIUS study is given in Figure 1. A total of 90,019 subjects were sent an invitation letter (and a reminder after 2 weeks) by mail. We were able to get a response from 55% either by response card or after a home visit by an ethnically matched interviewer. Of those, 24,789 agreed to participate (participation rate of 45%). After a positive response, participants received a confirmation letter of the appointment for the physical examination, including a digital or paper version of the questionnaire (depending on the preference of the subject). Participants who were unable to complete the questionnaire themselves were offered assistance from a trained ethnically matched interviewer.

For the current study, we used baseline data of all participants in whom questionnaire data as well as data from the physical examination were available (*n* = 22,165). We excluded those of Javanese Surinamese (*n* = 233) or unknown Surinamese (*n* = 267) origin due to small numbers and we also excluded those with another/unknown ethnic origin (*n* = 48). Participants who had missing data on the presence of diabetes (*n* = 113) were also excluded. Finally, participants who reported an age of onset of their diabetes before the age of 30 years and to have started insulin injections immediately after being diagnosed (*n* = 21) were also excluded, because these participants are very likely to have type 1 diabetes. Therefore, data of 21,483 participants were available for analyses, including 4,541 Dutch, 3,032 South-Asian Surinamese, 4,109 African Surinamese, 2,323 Ghanaian, 3,591 Turkish, and 3,887 Moroccan origin participants.

### 2.2. Ethnicity

Ethnicity was defined according to the country of birth of the participant as well as that of his/her parents, which is currently the most widely accepted and most valid assessment of ethnicity in Netherlands [21]. Specifically, a participant is considered to be of non-Dutch ethnic origin if he/she fulfils either of the following criteria: (1) he or she was born abroad and has at least one parent born abroad (first generation) or (2) he or she was born in Netherlands but both his/her parents were born abroad (second generation). Of the Surinamese immigrants in Netherlands, approximately 80% are of either African or South-Asian origin. Surinamese subgroups were classified according to self-reported ethnic origin. Participants were considered to be of Dutch origin if the person and both parents were born in Netherlands.

### 2.3. Diabetes

Fasting blood samples were used to determine the concentration of glucose by spectrophotometry, using hexokinase as primary enzyme (Roche Diagnostics, Japan). Participants were asked to bring their prescribed medications, which were coded according to the Anatomical Therapeutic Chemical (ATC) classification [22]. Diabetes mellitus was considered to be present if the participants' fasting glucose level was ≥7.0 mmol/l, if the participant was using glucose-lowering medication, and/or if the participant self-reported to have been diagnosed with diabetes by a health care professional. Among those having diabetes, awareness was defined as self-reported diagnosis of diabetes. Medical treatment was defined as the use of glucose-lowering medication. Among those treated with medication, diabetes control was defined as HbA1c levels ≤53 mmol/mol (7.0%) [23].

### 2.4. Covariates

Information on socioeconomic status (level of education) and lifestyle factors (smoking, alcohol use, and physical activity) was obtained by questionnaire. Educational level was based on the highest qualification attained, either in Netherlands or in the country of origin, and it was categorized into four groups: (1) never been to school or elementary schooling only, (2) lower vocational schooling or lower secondary schooling, (3) intermediate vocational schooling or intermediate/secondary schooling, or (4) higher vocational schooling or university. Alcohol intake in the past 12 months (yes/no) and current smoking status (yes/no) were obtained. Habitual physical activity was measured with questions about the time spent on several activities during a normal week in the past few months using the Short Questionnaire to Assess Health-Enhancing Physical Activity (SQUASH) [24]. Participants were categorized as adherent (yes/no) to the Dutch guideline for physical activity when the sum of the number of days per week for each moderate- and high-intensity activity lasting at least 30 min was greater than or equal to five. Weight, height, waist circumference, and hip circumference were measured, and body mass index (BMI) and waist-to-hip ratio (WHR) were calculated, as described before [25].

### 2.5. Statistical Analyses

Characteristics of men and women in the different ethnic groups were described by means with standard deviations (continuous variables) or percentages (categorical variables). The prevalence of diabetes was calculated, stratified by ethnicity, sex, and 10-year age groups. Then, logistic regression analyses were performed to study ethnic differences in the prevalence of diabetes, adjusted for age and sex. Effect modification by sex was tested, by adding interaction terms to the regression models. Because of significant differences between men and women (*p* < 0.05), all further analyses were performed stratified by sex. We additionally adjusted for well-known risk factors ((abdominal) obesity, health-related behaviors, and education) by adding these variables to the regression models. Finally, we performed logistic regression analyses to study ethnic differences in awareness (among those with diabetes), medical treatment (among those with diabetes as well as among those aware of diabetes only), and control (among those receiving glucose-lowering medication), adjusted for age. Statistical analyses were performed using IBM SPSS Statistics version 23.

## 3. Results

Among both men and women, the Turkish and Moroccan groups were about 5 years younger than the other ethnic groups (Table 1). The Dutch participants were higher educated as compared with all other ethnic minority groups. Large differences in health-related behavior were found between the different ethnic groups. For example, smoking rates were particularly low among Ghanaian participants, whereas alcohol use was particularly low among Moroccan participants. All ethnic minority groups had higher BMI and WHR as compared with the Dutch participants. The unadjusted prevalence of diabetes, as well as known diabetes (self-reported), was higher among all ethnic minority groups as compared with Dutch participants.

**Figure 2:**
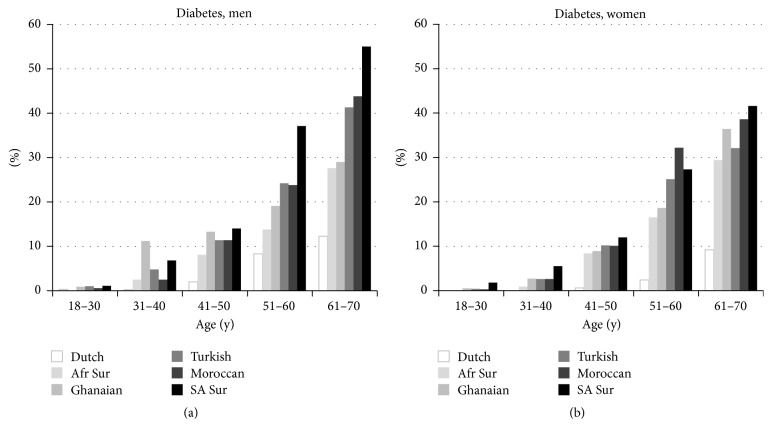
(a) The prevalence of diabetes by ethnicity, sex, and age groups (men). Afr Sur, African Surinamese; SA Sur, South-Asian Surinamese. (b) The prevalence of diabetes by ethnicity, sex, and age groups (women). Afr Sur, African Surinamese; SA Sur, South-Asian Surinamese.

In all ethnic groups, the prevalence of diabetes increased with age (Figure 2). The prevalence of diabetes among ethnic minority groups was much higher than that among Dutch participants, and this difference was already apparent and significant at the age of 31–40 years. The prevalence of diabetes among ethnic minority groups within a certain age group was comparable with the prevalence of diabetes among Dutch participants who were about 2 decades younger. For example, prevalence of diabetes in the oldest age group (61–70 years) among Dutch participants was similar to the prevalence among ethnic minority groups who were 41–50 years old (both men and women). Similarly, the prevalence of diabetes in the 51–60-year-old Dutch participants was similar to the prevalence among ethnic minority groups aged 31–40 years.

The ethnic differences in the prevalence of diabetes were also significant after adjustment for age, in both men and women (Table 2). Among men, ethnic minority groups had 3 to 8 times higher odds to have diabetes as compared with Dutch participants, and ethnic minority women had 6 to 12 times higher odds compared to Dutch women. Even after adjustment for conventional risk factors for diabetes (i.e., BMI, WHR, alcohol intake, smoking, physical activity, and educational level), ethnic minority groups had 3 to 5 times higher odds to have diabetes as compared with Dutch participants (data not shown).

Among all ethnic minority groups, the odds of being aware of diabetes were higher as compared with Dutch participants, though not statistically significant for Ghanaian women (Table 3). The awareness of diabetes was 70–80% among ethnic minority men, compared with 60% among Dutch men. Among women, the awareness was 80–90%. The odds of receiving medical treatment for diabetes were also higher in all ethnic minority groups as compared with Dutch participants. However, when restricting the medical treatment analyses to those aware of their diabetes, the odds of receiving medical treatment did not significantly differ between ethnic groups, except that African Surinamese and South-Asian Surinamese men had significantly higher odds of receiving medical treatment as compared with Dutch participants.

Among those treated with glucose-lowering medication, all ethnic minority men had significantly lower odds of controlled HbA1c levels as compared with Dutch men (control rates of 37.4–45.6% versus 67.4%, resp., Table 3). This could not be explained by the type of diabetes medication (oral or insulin), as the percentage of those using oral medication/insulin did not substantially differ between the Dutch and the other ethnic groups (94% using oral and 23% using insulin). Also, the age of onset of diabetes and diabetes duration among Dutch men were similar to the other ethnic groups (data not shown). In women, there were no significant ethnic differences in glycemic control (control rates ranging from 38.2 to 52.9%), while Dutch women relatively more often used insulin medication (35% versus 22–28% in the ethnic minority groups) and had the highest age of onset of diabetes and the longest diabetes duration compared with the other ethnic groups (data not shown).

## 4. Discussion

Already at young age, the prevalence of type 2 diabetes was higher among all ethnic minority groups as compared with the Dutch host population. Our results suggest a 20-year earlier onset of diabetes among the ethnic minority groups than that among the Dutch participants. Awareness and treatment rates of diabetes were significantly higher among ethnic minority groups compared with Dutch participants. If aware, all ethnic groups had similar odds of receiving medical treatment. Among those treated with glucose-lowering medication, despite high levels of awareness, only 37 to 53% had their HbA1c levels on target, except for Dutch men (67%). In women there were no significant ethnic differences in glycemic control.

Our finding of a higher prevalence of diabetes among ethnic minority populations as compared to the host population, even after adjustment for conventional diabetes risk factors, was consistent with several previous studies [8–13]. Our results also confirm previous smaller studies [11, 15, 17, 19] that suggested that diabetes starts to develop at a much younger age in ethnic minority groups as compared to Dutch participants. These findings suggest that case finding for diabetes should possibly start at younger age among these ethnic minority groups than that among the general population. The ADA suggests screening from the age of 45 years in overweight individual and suggests screening before the age of 45 years in overweight individuals who are of “high risk race/ethnicity” [23]. However, which ethnic groups, or from what age, is not specified. Guidelines in the UK suggest screening from the age of 25 years among South-Asians only [26]. Current Dutch general practice standards also suggest screening from the age of 45 years, and from the age of 35 in South-Asians, if a risk factor for diabetes (e.g., overweight or family history of diabetes) is present. Because the prevalence of diabetes among ethnic minority groups at age 31–40 years is similar to or even higher than the prevalence among Dutch participants at the suggested screening age of 45 years (41–50 years), our results suggest that the age limit should be lowered for African, South-Asian, Turkish, and Moroccan minority groups, rather than for South-Asians only.

Consistent with previous European studies [14–16], we did not find a lower diabetes awareness among ethnic minority groups as compared with the Dutch host population. US studies reported a lower awareness among ethnic minority groups [5, 6] and suggested this to be due to a lower percentage of ethnic minority people with health insurance, resulting in lower access to health care [6]. In Netherlands, a standard health insurance is legally obliged, which may explain the comparable or even higher awareness among the ethnic minority groups compared with Dutch participants in our study. These findings are consistent with recent findings on a higher hypertension awareness among ethnic minority groups compared with the Dutch host population in Netherlands [27]. Some studies have also suggested that ethnic minority groups are less likely to be prescribed lipid lowering and antihypertensive drugs compared with the host population [19, 28]. However, we found that levels of medical treatment with glucose-lowering agents were similar, or even higher, among ethnic minority groups. This, again, is probably due to the equal access to health care in Netherlands and is also consistent with recent findings on similar treatment rates among different ethnic groups in Netherlands [27].

Ethnic differences in glycemic control have previously been found in both US and UK studies, with lower control among ethnic minority groups [5, 17–19, 29]. Ethnic differences in control have been suggested to be due to poorer patient concordance (health literacy, poorer care standards), poorer quality of care, or lower response to diabetic agents among ethnic minority groups. In our study, despite equal access to health care, we found poorer glycemic control among ethnic minority men compared with Dutch men, which could not be contributed to differences in type of medication, diabetes duration, or age of onset of diabetes. Among women, there were no ethnic differences in glycemic control, and the level of control among women was similar to that of ethnic minority men. It is unclear why Dutch men showed a much higher level of glycemic control compared to all the other ethnic groups and compared with women. For hypertension control, control rates among men were consistently lower or similar when compared to women, in all ethnic groups (including Dutch) [27]. For glycemic control we observe the same gender difference, except for Dutch men. Regardless of ethnicity, however, the majority of medically treated patients did not have their HbA1c levels on target, suggesting that efforts to improve glycemic control should not only include ethnic minority groups. Noncompliance to treatment, lack of understanding of the disease, type of medication, or poor quality of health care professionals could all be involved in explaining these low glycemic control levels and require further investigation.

A limitation of our study is that fasting glucose was only measured on one single occasion. Therefore, there may have been some misclassification, and the prevalence of newly detected diabetes may have been somewhat overestimated, thereby affecting the estimation of awareness levels. However, this applies to all ethnic groups and if measured twice this would likely have led to even higher estimated awareness. Another limitation of our study is that we only studied treatment by glucose-lowering medication, whereas lifestyle interventions (either alone or in combination with medical treatment) may also help to obtain glycemic control. Of the different possible interventions, however, one would expect that medical treatment would have the highest impact on lowering glucose levels, whereas even in this group glycemic control was disappointing.

## 5. Conclusion

South-Asian, African, Turkish, and Moroccan ethnic minority groups had a much higher prevalence of type 2 diabetes compared with the Dutch host population. The high prevalence among these ethnic minority groups already occurred at young age, suggesting that the age limit for case finding should possibly be lowered for all these ethnic groups. Given the high prevalence of diabetes among young ethnic minority groups and the low levels of glycemic control in general, adequate detection and treatment deserve increased attention, particularly among ethnic minority groups. Further research should focus on the causes of poor glycemic control among those using glucose-lowering medication.

## Figures and Tables

**Table 1:** Characteristics of the study population, by ethnicity and sex.

	Dutch	South-Asian Surinamese	African Surinamese	Ghanaian	Turkish	Moroccan
*Men*						
*N*	2080	1364	1598	901	1624	1505
Age (y)	46.9 ± 13.8	44.8 ± 13.6	48.2 ± 12.9	46.9 ± 11.5	40.9 ± 12.1	42.1 ± 12.8
Education (%)						
1 (lowest)	3.4	13.0	6.6	15.9	24.6	25.7
2	13.6	32.4	40.8	45.9	30.8	21.8
3	23.4	30.7	34.0	29.4	28.5	33.4
4 (highest)	59.6	23.9	18.6	8.9	16.0	19.1
Current smoking (% yes)	26.2	39.8	42.9	7.6	41.1	26.3
Alcohol (% yes)	93.7	67.1	79.2	53.8	34.9	12.9
Achieving PA norm (% yes)	73.0	58.0	69.0	62.5	50.0	56.1
BMI (kg/m^2^)	25.2 ± 3.8	25.8 ± 4.2	26.3 ± 4.4	26.7 ± 3.8	27.9 ± 4.4	26.7 ± 4.0
WHR	0.94 ± 0.07	0.97 ± 0.08	0.93 ± 0.07	0.94 ± 0.07	0.96 ± 0.07	0.94 ± 0.07
Diabetes^†^ (% yes)	5.0	21.5	11.5	14.9	11.3	12.0
Known diabetes^∞^ (% yes)	3.0	17.6	8.2	12.4	8.3	9.8
*Women*						
*N*	2461	1668	2511	1422	1967	2382
Age (y)	45.6 ± 14.2	46.1 ± 13.2	47.8 ± 12.3	43.4 ± 10.7	40.0 ± 12.2	39.4 ± 12.9
Education (%)						
1 (lowest)	3.2	15.7	5.0	37.0	37.5	34.6
2	14.8	34.1	32.7	36.3	20.0	15.5
3	20.7	27.9	36.6	22.3	28.6	33.3
4 (highest)	61.3	22.3	25.8	4.5	14.0	16.6
Current smoking (% yes)	23.3	19.0	24.5	2.6	29.2	5.3
Alcohol (% yes)	88.8	47.4	62.1	43.5	12.7	3.9
Achieving PA norm (% yes)	77.8	49.6	56.5	47.3	35.1	40.9
BMI (kg/m^2^)	24.4 ± 4.5	26.7 ± 5.3	28.8 ± 5.9	29.6 ± 5.3	29.1 ± 6.6	28.1 ± 5.8
WHR	0.84 ± 0.08	0.90 ± 0.08	0.88 ± 0.08	0.88 ± 0.08	0.87 ± 0.09	0.86 ± 0.09
Diabetes^†^ (% yes)	2.4	17.7	12.1	9.6	9.3	10.8
Known diabetes^∞^ (% yes)	1.9	16.0	10.5	7.6	8.1	9.3

Data are mean with standard deviation or percentages. BMI = body mass index. WHR = waist-to-hip ratio.

^†^Diabetes based on self-report, fasting glucose ≥ 7 mmol/l, and/or use of glucose-lowering medication.

^∞^Diabetes based on self-report.

**Table 2:** Ethnic differences in the prevalence of diabetes^†^ by sex, adjusted for age.

	*Men*				*Women*			
	*n/N*	%	OR	95% CI	*n/N*	%	OR	95% CI
Dutch	105/2080	5.0	1.0 (ref)		58/2461	2.4	1.0 (ref)	
South-Asian Surinamese	293/1364	21.5	8.0	6.3–10.4	296/1668	17.7	12.2	9.0–16.5
African Surinamese	183/1598	11.5	2.6	2.0–3.4	303/2511	12.1	6.5	4.8–8.7
Ghanaian	134/901	14.9	4.6	3.5–6.1	136/1422	9.6	8.9	6.4–12.4
Turkish	183/1624	11.3	5.3	4.1–7.0	183/1967	9.3	10.5	7.6–14.5
Moroccan	181/1505	12.0	4.8	3.7–6.3	258/2124	10.8	12.3	9.0–16.7

^†^Diabetes based on self-report, fasting glucose ≥ 7 mmol/l, and/or use of glucose-lowering medication.

**Table 3:** Ethnic differences in diabetes awareness, treatment, and control among those with diabetes by sex, adjusted for age.

	Awareness (among all DM cases)^¥^			Medical treatment (among all DM cases)^¥^			Medical treatment (among those aware)			Control of HbA1c levels (among those medically treated) ^$^		
	*n/N*	%	OR (95% CI)	*n/N*	%	OR (95% CI)	*n/N*	%	OR (95% CI)	*n/N*	%	OR (95% CI)
*Men*												
Dutch	63/105	60.0	Ref	44/105	41.9	Ref	44/63	69.8	Ref	29/43	67.4	Ref
SA Sur	239/291	82.1	3.8 (2.3–6.2)^*∗*^	212/293	72.4	4.8 (3.0–7.9)^*∗*^	199/239	83.3	2.6 (1.4–5.1)^*∗*^	80/208	38.5	0.3 (0.2–0.6)^*∗*^
African Sur	130/183	71.0	1.9 (1.1–3.1)^*∗*^	112/183	61.2	2.6 (1.6–4.3)^*∗*^	107/130	82.3	2.3 (1.1–4.6)^*∗*^	48/109	44.0	0.4 (0.2–0.8)^*∗*^
Ghanaian	110/132	83.3	4.8 (2.6–9.0)^*∗*^	69/134	51.5	2.3 (1.4–4.0)^*∗*^	63/110	57.3	0.8 (0.4–1.5)	31/68	45.6	0.4 (0.2–1.0)^*∗*^
Turks	134/182	73.6	2.7 (1.6–4.7)^*∗*^	100/183	54.6	2.8 (1.7–4.7)^*∗*^	94/134	70.1	1.5 (0.8–3.0)	37/99	37.4	0.3 (0.1–0.7)^*∗*^
Moroccan	147/181	81.2	3.7 (2.1–6.5)^*∗*^	107/181	59.8	2.8 (1.7–4.6)^*∗*^	104/147	70.7	1.3 (0.7–2.6)	41/107	38.3	0.3 (0.2–0.7)^*∗*^
*Women*												
Dutch	46/58	79.3	Ref	34/58	58.6	Ref	32/46	69.6	Ref	16/34	47.1	Ref
SA Sur	264/295	89.5	2.9 (1.4–6.3)^*∗*^	219/296	74.0	2.9 (1.6–5.2)^*∗*^	202/264	76.5	2.0 (1.0–4.1)	83/217	38.2	0.7 (0.4–1.5)
African Sur	262/301	87.0	2.1 (1.0–4.5)^*∗*^	239/303	78.9	3.5 (1.9–6.3)^*∗*^	225/262	85.9	3.5 (1.7–7.3)^*∗*^	119/234	50.9	1.2 (0.6–2.5)
Ghanaian	106/136	77.9	1.4 (0.6–3.0)	91/136	66.9	2.4 (1.2–4.7)^*∗*^	79/106	74.5	2.1 (1.0–4.7)	47/87	54.0	1.4 (0.6–3.3)
Turks	157/182	86.3	2.4 (1.1–5.4)^*∗*^	116/183	63.4	2.0 (1.1–3.8)^*∗*^	104/157	66.2	1.4 (0.7–3.0)	43/112	38.4	0.8 (0.3–1.7)
Moroccan	220/257	85.6	2.2 (1.0–4.6)^*∗*^	172/258	66.7	2.1 (1.2–3.9)^*∗*^	160/220	72.7	1.8 (0.9–3.7)	80/170	52.9	1.1 (0.5–2.3)

DM, diabetes mellitus; OR, odds ratio; CI confidence interval; SA Sur, South-Asian Surinamese; African Sur, African Surinamese.

Awareness: those with diabetes who self-reported to have been diagnosed with diabetes; Medical treatment: those who take glucose-lowering medication; Control: those taking glucose-lowering medication who have HbA1c < 53 mmol/mol (7.0%).

^*∗*^ *p* < 0.05.

^¥^Number of all DM cases (denominator) may slightly differ due to a few missing cases on awareness.

^$^Number of medically treated participants (denominator) may vary due to few missing glucose or HbA1c levels.
